# Acute respiratory distress after exposure to chlorine dioxide-based disinfectant

**DOI:** 10.1093/occmed/kqac078

**Published:** 2022-10-18

**Authors:** Erlend Hassel, Hans Thore Smedbold, Hilde Brun Lauritzen

**Affiliations:** Department of Occupational Medicine, St Olavs Hospital, Trondheim University Hospital, Trondheim, Norway; Department of Public Health and Nursing, Faculty of Medicine and Health Sciences, Norwegian University of Science and Technology, Trondheim, Norway; Norwegian Armed Forces Occupational Health Service, Trondheim, Norway; Department of Occupational Medicine, St Olavs Hospital, Trondheim University Hospital, Trondheim, Norway; Department of Public Health and Nursing, Faculty of Medicine and Health Sciences, Norwegian University of Science and Technology, Trondheim, Norway; Department of Occupational Medicine, St Olavs Hospital, Trondheim University Hospital, Trondheim, Norway; Department of Public Health and Nursing, Faculty of Medicine and Health Sciences, Norwegian University of Science and Technology, Trondheim, Norway

## Abstract

A hospital cleaner developed acute respiratory distress after working with a chlorine dioxide-based disinfectant. The content of chlorine dioxide in the product is below the limit that would require the product to be labelled as hazardous to health, but we show with a simple estimation that the relevant threshold limit values for chlorine dioxide in the working atmosphere may be exceeded under normal use of the product. This may have implications for risk assessment of the use of such chlorine dioxide-based disinfectants and may warrant stricter regulations for labelling these products.

## Teaser

Chlorine dioxide has become a widely used disinfectant during the COVID-19 pandemic. Even at very low concentrations, it is a fast-acting broad-spectrum surface disinfectant. Chlorine dioxide has irritant properties, but since the concentration used is so low, the product has been considered a safe alternative to many other disinfectants. However, this case report suggests that even though the concentration of disinfectant fluid is very low, there may still be serious health risks associated with its use.

**Key learning points**

What is already known about this subject:

- During the COVID-19 pandemic chlorine dioxide has emerged as a widely used surface disinfectant.
- Chlorine dioxide has been marketed as the ideal surface disinfectant: efficient against near all pathogenic microorganisms, fast-acting, non-toxic and safe to use.
- Chlorine dioxide gas is a known irritant, but the concentration in the product used for surface disinfection is below the limit requiring the product to be labelled as potentially hazardous to health.

What this study adds:

- Even though the chlorine dioxide concentration in the aqueous solution is very low, when the gas is released during use, the concentration in the working atmosphere may exceed relevant occupational exposure levels.
- This case raises concern that normal use of these products may cause respiratory health damage.

What impact this may have on practice or policy:

- The current legislation should be revised so that such products are required to be marked according to their potential health hazard.
- Before adopting this chemical for surface disinfection purposes, careful assessment of the risk for health damage is advised, including measurements of chlorine dioxide concentrations in the working atmosphere.
- In healthcare settings, the health hazard for patients should also be considered. Patient rooms should be adequately ventilated before they are used after having been disinfected with chlorine dioxide.

## Background

Recently, aqueous solution of chlorine dioxide gas has emerged as a commonly used disinfectant for both surfaces and equipment in various industries and in healthcare settings. Even though chlorine dioxide gas is a potent respiratory irritant, the typical concentration used in disinfectant solutions of chlorine dioxide, is below the levels requiring the product to be marked as hazardous to health according to the European Classification, Labelling and Packaging (CLP) regulation [1]. Here we present a case history of a hospital cleaner being admitted to the emergency department with acute respiratory distress following repeated exposure to chlorine dioxide disinfectant.

## Case presentation

A hospital cleaner in her early twenties was admitted to the emergency department with acute respiratory distress following an intensive period of work with repeated exposure to chlorine dioxide disinfectant.

## Investigations

On examination, she was found to be clinically obstructive with tachypnoea, tachycardia, expiratory and inspiratory wheezing, and prominent coughing. She had sinus tachycardia, otherwise normal ECG, normal chest x-ray and normal blood tests, including infection markers.

Shortly after she started working with chlorine dioxide disinfectant, the patient experienced sore throats and airways, nausea and headaches when applying the disinfectant. She gradually developed shortness of breath and wheezing related to her work exposure. In her experience, there was a clear connection between her symptoms and the use of chlorine dioxide disinfectant at work. In the beginning, she experienced the symptoms only during and shortly after exposure, but after a few months, she felt that the symptoms became more persistent, and she did not experience full remission between work shifts. Her symptoms were most pronounced when she got home after a shift. As she felt that lying down further exacerbated her difficulty breathing, she often slept sitting in a chair after having worked a night shift. After having worked four consecutive night shifts, she experienced respiration-dependent chest pain, chest tightness, coughing, wheezing, and difficulty breathing. She contacted the out-of-hours medical service, which led to her being admitted to the emergency department.

As a child, she had been medically treated for suspected asthma up to the age of two or three years. After that, no asthmatic symptoms until the current events. She reported a history of allergic symptoms with conjunctivitis and nasal obstruction after exposure to pollen and animal hair, but no asthmatic reactions to any exposures.

The patient started working as a hospital cleaner about six months prior to the admission. She mostly worked shifts in the emergency department, cleaning and disinfecting patient rooms. She reports cleaning and disinfecting up to 17 patient rooms per shift, which lasted 7–9 h. In the first few months, she used a potassium peroxymonosulphate-based disinfectant and experienced no work-related symptoms. After these initial months, the type of disinfectant was changed to a chlorine dioxide-based product. She used about 1 l of 200 ppm or 0.02% aqueous chlorine dioxide solution per room. She moistened microfiber cloths with the solution and then used these cloths to wipe off surfaces. For cleaning the patient’s bed and floor, she applied the solution directly with a squeeze bottle and then wiped it off. After wiping, a liquid-film should remain on the surface left to act on the surface for at least 2 min.

## Treatment

Upon admission, her condition was considered to be an acute exacerbation of asthma. She was given anti-obstructive treatment with salbutamol and ipratropium bromide on vaporizer and corticosteroids. Her condition gradually improved over the course of several hours and she was discharged the following day. She was prescribed anti-inflammatory treatment with inhaled corticosteroids, and she was told that she should avoid further exposure to chlorine dioxide disinfectant.

## Outcome and follow-up

In the period after the hospital admission, she avoided working with chlorine dioxide and her symptoms gradually resided, even though she stopped taking inhalation corticosteroids after a few weeks. During the following year, she still experienced symptoms of irritation in the throat and chest when exposed to chlorine dioxide. If she worked with chlorine dioxide over several days, she again experienced wheezing and shortness of breath. About eighteen months after the acute admission, she underwent spirometry that showed normal dynamic lung volumes and a methacholine challenge test with no sign of bronchial hyperreactivity with a PD20 of 1330 μg methacholine. At this time, she had been away from exposure to chlorine dioxide for at least one month and experienced no symptoms.

After several other hospital cleaners reported airway irritation when working with chlorine dioxide, the hospital suspended the use of chlorine dioxide disinfectant until further examinations due to concerns about employee and patient safety.

## Discussion

**Figure 1.**
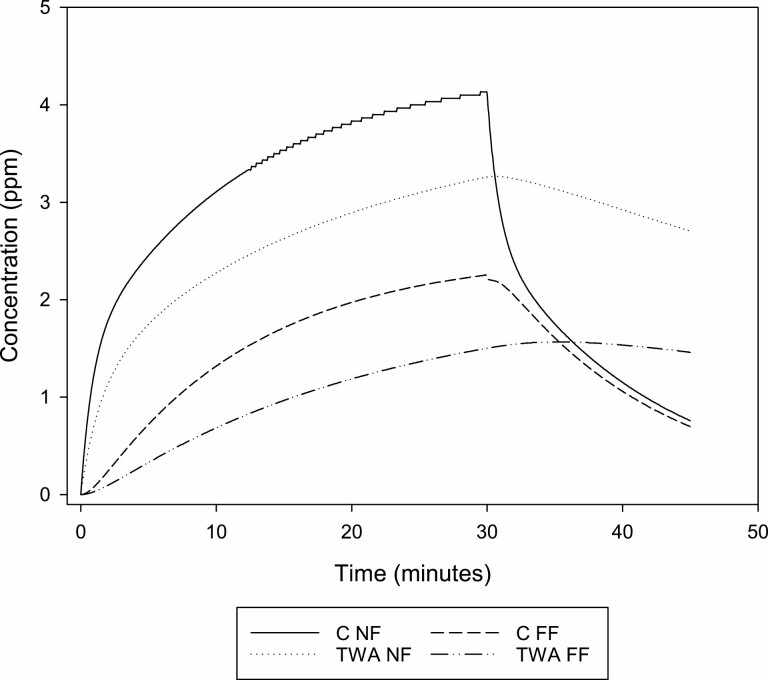
Exposure estimate chlorine dioxide based on the case report. NF = Near Field exposure, FF = Far Field exposure, C=Concentration, TWA=time weighted average.

Chlorine dioxide gas is a potent respiratory irritant, and the American Conference of Governmental Industrial Hygienists (ACGIH) has proposed a threshold limit value—ceiling (TLV-C) of 0.1 ppm in the working atmosphere due to the potential for respiratory tract irritation and pulmonary oedema [2]. Even though the concentration of chlorine dioxide in the disinfectant solution is relatively low, an estimation based on the described use and some theoretical assumptions shows that the patient may have been exposed to concentrations far above this threshold limit value, see time–history graph of estimated concentration in Figure 1 applying a Near Field–Far Field model [3]. This estimation shows a maximum concentration of 4 ppm and a 30-min mean of 3 ppm. The assumptions in this model are that 1 l of 200 ppm chlorine dioxide solution is used to clean a room with a 40 m^3^ volume over 30 min. It is assumed that all the chlorine dioxide gas in the solution is released as the solution evaporates and that the gas is evenly distributed in the room volume. The room is assumed to be ventilated with five air changes per hour. This shows that even though the product has a concentration below the threshold requiring it to be marked as potentially hazardous to health according to the European CLP regulation [1], the concentrations in the working atmosphere of cleaning personnel may greatly exceed the levels considered to be safe.

Our evaluation of this case history is that the patient’s symptoms most likely represent an irritant asthmatic reaction caused by repeated exposure to chlorine dioxide gas in concentrations that likely were above the relevant health-based threshold limit values. Her symptoms show a tight-linked relation with exposure to the disinfectant. Unfortunately, spirometry or other investigations objectively demonstrating airway reactions were not performed in the symptomatic phase of her condition. Differential diagnoses for the acute exacerbation leading up to the hospital admission are mainly infection or an allergic reaction. Infection is considered not to be likely due to the lack of fever or other clinical signs of infection, and the lack of infection markers in the blood. Allergic reaction is considered less likely because no plausible allergic agents have been identified. Infection or allergic reaction cannot explain the airway irritation she experienced during repeated exposures to chlorine dioxide.

This case report presents new information on a possible health hazard associated with the use of chlorine dioxide solutions for surface disinfection in an occupational setting. This health hazard is not currently reflected in the product’s Material Safety Data Sheet, as the concentration of the gas in the solution is below the level that requires it to be marked as potentially hazardous to health according to European CLP regulation. We show with a simple estimation that there is a potential exposure for chlorine dioxide gas well above relevant occupational exposure limit values during surface disinfection with 200 ppm chlorine dioxide solutions. Before adopting this chemical for surface disinfection purposes, we advise careful assessment of the risk for health damage. Preferably, these risk assessments should include measurements of chlorine dioxide concentrations in the working atmosphere. In hospital settings, also the potential health hazard for patients should be considered.
